# *Lactobacillus gasseri* CECT 30648 shows probiotic characteristics and colonizes the vagina of healthy women after oral administration

**DOI:** 10.1128/spectrum.00211-25

**Published:** 2025-08-07

**Authors:** Marta Perez, Eva Armengol, Antonio Del Casale, Ilenia Campedelli, Ana Aldea-Perona, Marta Pérez Otero, Maria Rodriguez-Palmero, Jordi Espadaler-Mazo, Pol Huedo

**Affiliations:** 1 R&D Department, AB-Biotics S.A. (Part of Kaneka Corporation) 602131 , Barcelona, Spain; 2 Basic Sciences Department, Universitat Internacional de Catalunya 602131 , Barcelona, Spain; 3 Open Innovation Department, MICROBION srl, Verona, Italy; 4 Clinical Pharmacology, Hospital del Mar 16770, Barcelona, Spain; 5 Hospital del Mar Medical Research Institute 16551, Barcelona, Spain; 6 Universitat Pompeu Fabra 16770, Barcelona, Spain; Universita degli Studi di Modena e Reggio Emilia, Modena, Italy

**Keywords:** vaginal lactobacilli, dysbiosis, antagonism activity, vaginal colonization, clinical study

## Abstract

Vaginal dysbiosis is linked to recurrent infections and reproductive complications. Probiotics may restore vaginal microbiota, but there is modest evidence to support vaginal colonization after oral administration. This work aimed to screen a vaginal lactobacilli collection (*n* = 45) and assess vaginal colonization of selected candidates. *Lactobacillus gasseri* CECT 30648 (Lg) and *Lactobacillus crispatus* CECT 30647 (Lc) showed excellent *in vitro* attributes, including antagonism against 10 urogenital pathogens, resistance to gastrointestinal tract and vaginal environment—including high concentrations of biogenic amines—and adhesion to vaginal epithelium. Forty-eight healthy women (18–45 years old) were randomly allocated to consume daily a capsule containing Lg (10^9^ CFU), a combination of Lg plus Lc (1.5 × 10^9^ CFU), or placebo for up to 18 days (between menses). Volunteers collected vaginal samples every 3 days. The primary endpoint was the presence of probiotic strains in vaginal swabs confirmed by strain-specific quantitative PCR. Secondary variables included vaginal microbiota composition and tolerability, among others. Probiotics were well tolerated. Lg but not Lc was detected in 55.9% of participants in active groups (9 in Lg and 10 in Lg + Lc) throughout the study (*P* = 0.005 aggregated probiotic groups vs placebo). The sum of relative abundances of non-lactobacilli genera was significantly reduced in the probiotic group after intervention (*P* = 0.047 vs day 0), and there was a positive transition toward lactobacilli-dominated community state types (*P* = 0.039 vs day 0). Overall, we show that *L. gasseri* CECT 30648 exerts a broad-spectrum antimicrobial activity and can colonize the vaginal tract of healthy women, thus showing potential to promote vaginal health. The trial was registered in clinicaltrials.gov (NCT05688397).

**IMPORTANCE:**

The use of probiotics to promote vaginal health is increasing because vaginal dysbiosis has been linked to numerous gynecological and reproductive complications. While vaginal administration of probiotics using devices or creams has been widely investigated, there is limited evidence supporting vaginal colonization of a probiotic administered orally. It is therefore imperative to perform extensive *in vitro* characterization to select a vaginal probiotic that can survive the gastrointestinal transit and effectively colonize the vaginal tract of consumers through the oral-gut-vaginal route. We have identified the strain *L. gasseri* CECT 30648, which shows great probiotic properties, including antagonism against several relevant urogenital pathogens, can colonize the vaginal tract of >55% of participants, and can modulate vaginal microbiota toward a lactobacillus-dominated status in a randomized controlled clinical trial in healthy premenopausal women. These results suggest that oral consumption of *L. gasseri* CECT 30648 might be effective in promoting vaginal health.

## INTRODUCTION

Vaginal health is closely linked to vaginal microbiota. A healthy vaginal ecosystem is typically dominated by one or multiple species of *Lactobacillus*, which are critical for the maintenance of vaginal homeostasis. Vaginal microbiota is classified depending on the dominant bacterial group in community state types (CSTs) (1). Four of them are dominated by one *Lactobacillus* sp., i.e., *Lactobacillus crispatus* (CST I), *Lactobacillus gasseri* (CST II), *Lactobacillus iners* (CST III), and *Lactobacillus jensenii* (CST V), and one is characterized by a more diverse microbiota dominated by other facultative and obligate anaerobic bacteria (CST IV), although later studies divided these groups into subcategories (2). Microbiotas dominated by *L. crispatus*, *L. gasseri*, and *L. jensenii* are generally associated with health and microbiota stability (3, 4). Such lactobacilli species protect the host by the production of antimicrobial compounds, regulation of pH levels, competition with pathogens for ecological niche, and modulation of immune responses (5).

Perturbations of the vaginal ecosystem are characterized by a reduction of lactobacilli abundance and an increase in microbiota diversity. Vaginal dysbiosis has been associated with an increased risk of suffering gynecological infections, including bacterial vaginosis (BV), aerobic vaginitis (AV), vulvovaginal candidiasis (VVC), and urinary tract infections (UTIs), as well as negative reproductive outcomes (6, 7). While BV is caused by the overgrowth of harmful anaerobes such as *Gardnerella vaginalis* or *Prevotella* spp. among others (8), the etiological agents of AV are intestinal aerobic bacteria such as *Escherichia coli*, *Klebsiella pneumoniae*, *Staphylococcus aureus*, group B streptococci, or *Enterococcus faecalis* (7). VVC occurs by the overgrowth of *Candida* spp. often related to antibiotic use, hormonal changes, or immune deficiency (9). UTIs are caused by the proliferation of bacteria in the urinary tract, typically uropathogenic *E. coli* but also staphylococci and streptococci. UTIs are associated with vaginal dysbiosis since the vagina (together with the gut) serves as a reservoir for pathogens that can migrate to the urinary tract (10). Several of the mentioned pathogens have also been linked to fertility issues, miscarriage, and preterm birth (3, 11, 12). Recently, *Fusobacterium* (at all taxonomic levels) has been associated with abortion (13), and the genus is linked to vaginal inflammation and preterm delivery (14).

Although antibiotic and antifungal treatments are available to treat vaginal infections, high rates of recurrence, together with increasing prevalence of resistant strains, make alternative therapies necessary. Probiotics, defined as live microorganisms conferring health benefits when administered in adequate amounts (15), have demonstrated efficacy in reducing vaginitis symptoms and recurrence rates (16). While the administration of probiotics via vaginal suppositories or creams has been investigated extensively, the exploration of oral probiotic supplementation as a means of modulating the vaginal microbiota remains relatively understudied (17). Oral administration offers advantages including ease of use, improved patient compliance, and potential systemic effects beyond the vagina. However, probiotic strains for oral consumption must withstand the harsh gastric environment and traverse a lengthy journey through the gastrointestinal tract before reaching the vaginal mucosa. Thus, such strains must be selected after a rational screening. Some studies have explored the impact of oral administration of probiotics in women with vaginal infections (18, 19), pregnant women (20, 21), and postmenopausal women (22). Nonetheless, most studies have used lactobacilli probiotics that are neither from vaginal origin nor belong to dominant vaginal species and, to our knowledge, any of the studies implemented a frequent sampling combined with sensitive strain-specific detection methods to evaluate probiotic migration to the vagina.

In this context, our study aimed to screen a collection of vaginal lactobacilli and select the two strains showing better potential to promote vaginal health. Through a subsequent clinical trial involving healthy women, our objective was to evaluate the colonization ability of selected probiotic strains. By comprehensively evaluating colonization dynamics and microbial interactions, our study seeks to pave the way for innovative approaches to gynecological care, empowering women with effective strategies for maintaining vaginal health and overall well-being.

## MATERIALS AND METHODS

### Microorganisms and growth conditions

Forty-five strains of the species *L. gasseri* (*n* = 20) and *L. crispatus* (*n* = 25) isolated from the vaginal tract of healthy women belonging to the bacterial collection of Kaneka Corporation were investigated in this study. Pathogenic strains used in the antimicrobial activity tests were obtained from international culture collections. Strain details and growth conditions are listed in Table S1.

### Growth capacity screening of vaginal lactobacilli

Fresh overnight bacterial suspensions were adjusted (optical density at 600 nm [OD_600nm_] = 0.05), inoculated in de Man, Rogosa and Sharpe (MRS), and incubated for 16 h in anaerobiosis at 37°C. Those strains showing an optical density less than 1 in at least two of the three replicates were discarded from further analysis.

### Antimicrobial activity against urogenital pathogens

Antagonistic activity of vaginal lactobacilli was determined by agar spot test against *G. vaginalis* DSM 4944 and *Prevotella bivia* DSM 2051. Overnight cultures of vaginal lactobacilli were used to inoculate MRS soft agar (0.9% agar) at 10% to create agar disks containing lactobacilli. Disks were incubated at 37°C for 24 h in an anaerobic atmosphere. A standardized suspension (10^8^ CFU/mL) of the target pathogen strain was spread onto nutrient agar plate, and the agar disks containing lactobacilli were deposited on top. After 48 h of incubation at 37°C, the plates were checked to evaluate the inhibition zone. The inhibitory halos were measured from the outer perimeter of the disks. Three replicates were performed. The antagonistic activity was expressed as −, no inhibition; ±, radius <1 mm; +, radius between 1 and 10 mm; ++, radius between 11 and 19 mm; +++, radius >20 mm.

Broth inhibition assay was performed to evaluate the inhibitory effect of lactobacilli strains on the growth of *Candida* spp. strains. Cell-free supernatants of 16 h cultures of lactobacilli in MRS were obtained by centrifugation and filtered through 0.22 µm filters. A volume of 2.5 mL of the supernatant was added to 2.5 mL of yeast extract peptone dextrose (YPD) medium. An overnight culture of the target strain was standardized to a cell concentration equal to McFarland 0.5, diluted 1:100, and finally used to inoculate the mixed broth medium described above. YPD medium without lactobacilli supernatant was used as control. The growth curve of the target microorganisms was monitored for 24 h, and final OD_600nm_ was used to calculate the growth inhibition rate in percentage as in De Gregorio et al. (23). Two replicates were performed.

Antagonistic activity against additional bacterial pathogens was tested by microplate growth inhibition assay following the protocol described previously (24) with slight modifications. Twofold concentrated (2×) fresh medium (MRS for probiotics and tryptic soy broth [TSB] or brain heart infusion [BHI] for pathogens) was inoculated (0.1%) with the preinoculum adjusted to OD_600nm_ = 0.16 and cultured for 16 h at 37°C (probiotics were incubated anaerobically and pathogens aerobically). Then, two different mixtures were performed in 50/50 proportions to obtain monocultures and co-cultures. Monocultures were obtained by mixing probiotic culture with TSB or BHI medium, and co-cultures were obtained by mixing probiotic culture with pathogen culture. Monocultures and co-cultures were further incubated for 24 h anaerobically. Cultures were then centrifuged, and supernatants were filtered (0.22 µm). An aliquot was adjusted to pH 5 except for *Streptococcus agalactiae* co-cultures that were adjusted to pH 6. Samples were stored at −20°C until analysis. The antimicrobial activity of monoculture and co-culture supernatants was tested as follows. The pathogen was grown in the corresponding pathogen liquid medium for 24 h. Pathogen cultures were adjusted to OD_600nm_ = 0.2 with 2× TSB or BHI media, and 100 µL was added to wells of a 96-well plate. One hundred microliters of crude and neutralized monoculture and co-culture supernatants was added to the wells. Plates were incubated aerobically or anaerobically by adding 20 µL of oil to the corresponding wells at 37°C, and OD_600nm_ was monitored for 24 h in a plate reader. At least two independent replicates were performed. Growth curves were obtained and area under the curve (AUC) was calculated and normalized with baseline optical density. Results are shown as the mean and standard deviation of the percentage of inhibition growth in relation to that of the control well.

### Resistance to simulated gastrointestinal and vaginal conditions

To study gastric stress resistance, overnight cultures were inoculated (1%) into simulated gastric solutions (per liter: NaCl 7.3 g, KCl 0.52 g, NaHCO_3_ 3.78 g, and pepsin 3 g) adjusted to pH 2.3 and to pH 3.0 with 1 N HCl. Bacterial suspensions were incubated at 37°C under anaerobic conditions for 30 min in pH 2 condition (simulating fast gastric passage) and for 90 min in pH 3 condition (slow gastric passage). To determine bile salt tolerance, overnight cultures were inoculated (1%) into MRS containing 0.25% (wt/vol) bile salts and incubated for 3 h under the same conditions. To assess resistance to vaginal conditions, overnight cultures were inoculated (1%) into simulated vaginal media [per liter: NaCl 3.51 g, KOH 1.40 g, Ca(OH)_2_ 0.222 g, bovine serum albumin 0.018, lactic acid 2 g, acetic acid 1 g, glycerol 0.16 g, urea 0.4 g, glucose 8 g, adjusted to pH 4.2 with 1 N HCl] and incubated at 37°C. Samples were collected at 0, 3, 6, and 24 h. All samples were seeded onto MRS agar and incubated at 37°C for 48 h in anaerobiosis. Three independent replicates were performed. Results are presented as mean and standard deviation of log CFU per milliliter.

Resistance to biogenic amines was investigated through growth curve experiments using 96-well plates. Overnight cultures were inoculated into MRS medium (final OD_600nm_ = 0.2) supplemented with tyramine or cadaverine at a range of concentrations found in the vagina of BV patients (25): 1,000, 600, 300, or 100 mg/L (Sigma, Spain). Optical density (OD_600nm_) was monitored for 24 h. Growth curves were obtained, and the AUC was calculated and expressed as percent growth compared to MRS control conditions. At least two independent replicates were performed.

### Quantification of biogenic amines and lactic acid biosynthesis

The ability to produce four biogenic amines through five biosynthetic routes—putrescine from agmatine and from ornithine, histamine from histidine, cadaverine from lysine, and tyramine from tyrosine—was investigated as previously described (24). Strains were cultured in MRS supplemented with 1 mM precursor for 24 h. Production of biogenic amines was assessed by ultra-high-performance liquid chromatography, as described in reference 26 at Instituto de Productos Lácteos de Asturias – Consejo Superior de Investigaciones Científicas (IPLA-CSIC) (Spain). Total lactic acid was measured in the supernatant of 72 h cultures in MRS by high-performance liquid chromatography (27) at University of Valencia (Spain). L-lactate isomer was quantified by an enzymatic kit (BioSystems, Spain). Three replicates were performed.

### Adhesion to HeLa cells

Adhesion capacity to vaginal epithelium was evaluated using the human vaginal epithelial HeLa cell line following the protocol described by Auger et al. (28) with minor modifications. HeLa cells were cultured in Dulbecco’s modified Eagle medium high glucose supplemented with 10% fetal bovine serum in 24-well plates at 37°C with 5% of CO_2_ until they reached at least 85% confluence. A standardized suspension of lactobacilli was added to HeLa cells at a multiplicity of infection of 5:1 (bacteria:cell), and the plate was incubated for 1 h. Then, the medium was removed, and the monolayer was washed. Cells with adhered bacteria were trypsinized, and the suspension was serially diluted, plated, and incubated according to “Rapporti Istisan 08/36” (29). Adhesion percentages were calculated as the log CFU of adhered bacteria relative to the log CFU of the inoculated bacteria. Four independent replicates were performed.

### *In silico* analyses

The genomes of *Lactobacillus gasseri* CECT 30648 (Lg) (KABP064) and *Lactobacillus crispatus* CECT 30647 (Lc) (KABP066) (30) were inspected for the presence of bacteriocins using the BAGEL4 web server (31). To gain insights into adhesion determinants, proteomes were examined for the presence of proteins containing cell wall anchor (gram_pos_anchor or LPxTG) domains and extracellular YSIRK signals (32), and the identified extracellular proteins were also screened for the presence of mucus-binding domains (muc_B2/mucBP/mucBP_2) through eggNOG mapper (33) and InterProt scan (34).

### Design of strain-specific primers

The genomes of *L. gasseri* CECT 30648 and *L. crispatus* CECT 30647 were aligned against a collection of *L. gasseri* and *L. crispatus* genomes (Table S1), and exclusive regions were identified using Basic Local Alignment Search Tool (BLAST) (35). An exclusive region of 554 bp was found in the genome of *L. crispatus* CECT 30647 in contig 9 positions 10,481–11,035. BLAST analyses of this region only produced one match (100% Id, 100% coverage) with *L. crispatus* strain L116 (GenBank accession number CP083393.1) in the 3′ region of locus UAY50261.1, which encodes for a hypothetical protein. An exclusive region of 499 bp was identified in the genome of *L. gasseri* CECT 30648 in contig 1 positions 88,972–89,471. BLAST analyses of this region found no significant similarities in the nr/nt National Center for Biotechnology Information database (consulted in September 2024). Based on detected exclusive regions, oligos were designed to have a Tm around 60°C and an amplicon length of around 120 bp (Table S3) using software Oligo 7 (36) and were synthesized by Biomers.net GmbH (Germany).

Oligo specificity was validated by quantitative PCR (qPCR) using Applied Biosystems 7500 Thermocycler and PowerUP SYBR Master Mix (Thermo Fisher, Spain) with the following program settings: 50°C 2 min; 95°C 10 min; 45× (95°C 15 s, 65°C 30 s, 72°C 30 s); 95°C 10 s; 60°C 1 min; 95°C 15 s; and 60°C 15 s against a collection of vaginal isolates of *L. gasseri* and *L. crispatus* (normalized to 100 ng/µL) using primers at a final concentration of 0.5 µM (Table S4).

Oligo sensitivity was validated by spiking vaginal swabs from the placebo group with decreasing concentrations of *L. gasseri* and *L. crispatus* DNA (Table S5). Samples with Ct lower than 36 were considered positives. Both oligo sets allowed the detection of up to 0.001 ng/µL of the targeted DNA. In all spike-in samples, blank samples were above 36 Ct. Representative melt curves of qPCR-positive and qPCR-negative controls and vaginal samples using primers LG4107_F1 and LG4107_R (Table S3) are shown in Fig. S1.

### Clinical trial

#### Study design and participant selection

**Fig 1:**
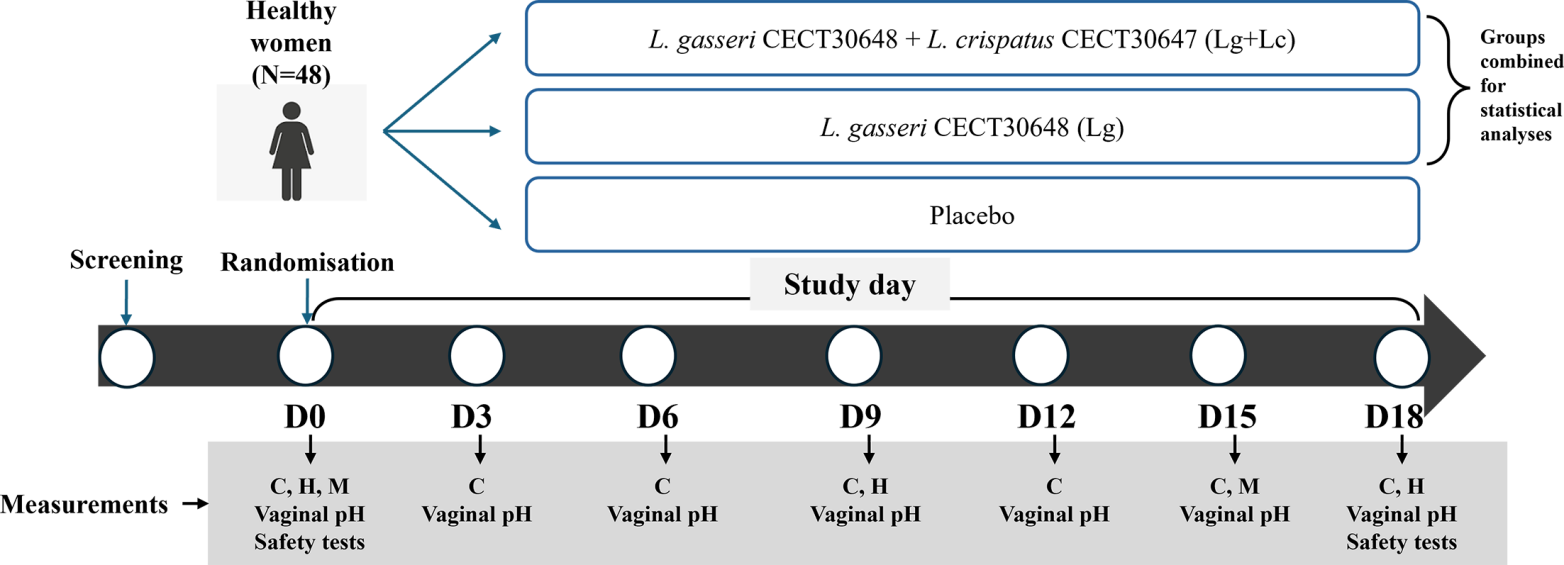
Schematic illustration of the clinical study. C, colonization detection by qPCR; H, hormonal levels in serum; M, microbiota composition of vaginal samples analyzed by 16S rRNA sequencing. The groups Lg+Lc and Lg were aggregated in the probiotic group for statistical purposes.

A prospective, randomized, double-blind, and placebo-controlled study with three parallel arms was conducted at the Clinical Research Unit of Hospital del Mar Research Institute (Barcelona, Spain) from 15 February to 30 June 2023. A schematic illustration of the study design is shown in Fig. 1.

Healthy premenopausal women aged 18–45 years were recruited voluntarily. Main exclusion criteria included active vaginal infection; irregular menstrual cycles; use of intrauterine devices; use of probiotics, antibiotics, or antifungals 1 month before study start; willingness to maintain sexual abstinence for 24 h before vaginal sampling; or any relevant medical condition. Additional exclusion criteria included self-report of inflammatory bowel disease, immunodeficiencies, diabetes, estrogen-dependent cancer, pancreatitis, chronic diarrhea or constipation, pregnancy (checked with a urine pregnancy test before inclusion) or lactation, use of immunomodulators or corticosteroids, antibiotics or systemic or vaginal antifungal drugs, substance abuse (checked with a urine drug test for cocaine, THC, morphine, amphetamines, and benzodiazepines before inclusion), atrophic vaginitis, recent use of spermicides or vaginal lubricants, short menstrual cycles, not being able to follow or understand study procedures, or any other incompatible conditions.

Participants were randomized in a 1.5:1.5:1.0 ratio to receive orally one capsule containing either (i) Lg 10^9^ CFU, (ii) a combination of *L. gasseri* CECT 30648 10^9^ CFU and *L. crispatus* CECT 30647 5 × 10^8^ CFU (Lg + Lc), or (iii) placebo with identical appearance to active capsules. All capsules contained maltodextrin as a carrier, magnesium stearate as an antiadherent, and hypromellose as a capsule coating and were produced by AB-Biotics, S.A. (Spain). Stability of study products was confirmed by CFU enumeration throughout the intervention period.

Participants were instructed to consume one capsule daily between menses, up to 18 days, defined according to average intermenstruation period. The randomization list was generated using randomly permuted blocks through specific software (Sealed Envelope Ltd. 2022) and kept confidential by the sponsor. Based on similar studies (37, 38) and considering the variability between menstrual cycles in women, we deemed it necessary to include a total of 48 participants, 18 participants in each probiotic group, and 12 participants in the control group.

The study comprised four on-site visits: screening, baseline visit (day 0), visit 3 (day 9), and final visit (day 18) and a total of four self-evaluation timepoints at visit 1 (day 3), visit 2 (day 6), visit 4 (day 12), and visit 5 (day 15). At the screening visit, informed consent was obtained from those women who met the selection criteria and agreed to participate. At the baseline visit, randomization was performed, and participants received instructions and kits for self-sample collection at home and were asked to abstain from sexual intercourse and the use of intravaginal products such as lubricants and spermicides for 24 h before sampling. Telephone calls were performed every 3 days to remind participants about product intake and obtention of vaginal samples and to ask for potential adverse events. For those women having menstruation prior to day 18, the last visit control was performed, and the last sample collected was considered the final sample.

#### Study outcomes

The primary endpoint of the study was the presence of *L. gasseri* CECT 30648 and *L. crispatus* CECT 30647 in vaginal swabs confirmed by specific qPCR. Secondary endpoints included vaginal pH, sexual hormone concentrations in serum, and vaginal microbiota composition. Digestive tolerability was assessed by a modified version of the Gastrointestinal Symptom Rating Scale (39) at baseline and at the final visit, with severity rated on a 4-point scale (0, no discomfort; 1, mild; 2, moderate; and 3, severe). At the final visit, the participants also completed the TSQM 1.4 questionnaire (40) to evaluate treatment satisfaction. As safety outcomes, blood analysis comprising complete blood cell count, hematology, routine biochemistry including glucose levels, renal function markers (urea, creatinine, and uric acid), liver function tests (aspartate aminotransferase, alanine aminotransferase, gamma-glutamyl transferase, bilirubin, and alkaline phosphatase), lipid profile, electrolytes, and urinalysis parameters (glucose, ketone bodies, bilirubin, urobilinogen, protein, and nitrites) were also performed at baseline and final visits. All analyses were conducted at the Laboratori de Referència de Catalunya using standard commercial assay kits. In addition, the number and type of adverse events were recorded throughout the study.

#### Sample collection and analysis

Participants collected two vaginal swabs at every visit and timepoint using self-collection devices for vaginal samples (OMR-130; OMNIgene, Canada). Presence of probiotic strains was analyzed in duplicated samples by strain-specific qPCR at all study timepoints. Vaginal pH was self-determined by participants at all timepoints using an applicator (Geratherm pH balance, Germany) according to manufacturer’s instructions. Serum hormone concentrations (total estradiol, progesterone, testosterone, dehydroepiandrosterone, follicle-stimulating hormone, and luteinizing hormone) were analyzed by electrochemiluminescent method (Cobas 8000, Roche) at days 0, 9, and 18.

DNA was extracted from vaginal swabs using a standardized protocol involving automated physical disruption via bead beating, along with chemical cell lysis using the DNeasy PowerLyzer PowerSoil Kit (Qiagen, Germany) following the manufacturer’s instructions (41). DNA samples were analyzed using qPCR assays employing strain-specific primers for *L. gasseri* CECT 30648 and *L. crispatus* CECT 30647 as indicated above. Technical triplicates were tested, and positive and negative controls were included. Analysis was performed using 7500 software v.2.3. Dissociation curves were examined to ensure specificity of amplification.

Vaginal microbiota composition was studied by 16S rRNA gene amplicon sequencing as described in Barba et al. (41) with slight modifications. The V3–V4 region of the 16S rRNA gene was amplified (forward 5′TCGTCGGCAGCGTCAGATGTGTATAAGAGACAGCCTACGGGNGGCWGCAG-3′, reverse 5′GTCTCGTGGGCTCGGAGATGTGTATAAGAGACAGGACTACHVGGGTATCTAATCC-3′). Mock community DNA was included as positive and quality controls for library preparation (Zymobiomics Microbial Community DNA; Zymo Research, Irvine, CA, USA). The sequencing library was prepared using Nextera XT v.2 (Illumina) according to the manufacturer protocol. PCR products were purified with SequalPrep normalization kit (Invitrogen, Thermo Fisher Scientific, Waltham, MA, USA) and pooled for sequencing. Sequencing was performed using Illumina Illumina MiSeq (2 × 300 bp reads) platform. Phylotype data were used to calculate the following alpha-diversity metrics: richness, Pielou’s evenness, and Shannon index. The phylotype and phylogenetic data were used to calculate beta-diversity unweighted and weighted Unifrac, Jaccard, and Bray-Curtis distances (42). Taxonomic assignment of amplicon sequence variants (ASVs) was performed using a Bayesian Classifier trained with SILVA v.138 database (i.e., 99% operational taxonomic unit [OTU] database) (43) using the qiime feature-classifier classify-sklearn method (44). *Lactobacillus* genus *sensu lato* was reassigned using BLAST v.2.12 (35) against in-house filtered SILVA database. Microbiota composition statistical analysis was performed as follows. Alpha-diversity comparisons were performed using a generalized linear mixed model (GLMM). The R package NBZIMM v.1.0 (45) was used for richness, and the R package betareg v.3.1-4 (46) was used for evenness. Beta-diversity distance matrix and ASV tables were used to calculate principal coordinates and construct ordination plots using Past 5 v5.2.2. The significance of groups in community structure was tested using two-way permutational multivariate analysis of variance. Differential abundance of taxa was tested using negative binomial GLMM. Significant threshold was set at 0.05.

CST classification was performed following France et al. (2) using main categories CST I, II, III, IV, and V due to the reduced number of participants. CST II and V were further grouped with CST I for statistical analysis. Alluvial plots were generated using RAWGraphs 2.0.

### Statistical analysis

*In vitro* data were analyzed by one-way analysis of variance followed by Dunnett’s post hoc test when indicated. Per protocol (PP) analyses were planned in the clinical study. Baseline demographic data were summarized using descriptive statistics (mean and standard deviation). The chi-squared test (with the N−1 correction for small numbers) was used to compare categorical variables between study groups, while the sign (exact) test was used to assess change in CSTs between CST I–II–V, CST III, and CST IV groups. Continuous variables were compared with Student’s *t*-test (unpaired for between-group comparisons and paired for within-group comparisons), or its non-parametric equivalents whenever residuals were not normally distributed. Statistical significance was set at *P* < 0.05, and analyses were performed with GraphPad PRISM.

## RESULTS

### Screening of vaginal lactobacilli and selection of potential probiotic strains

The strain selection process is illustrated in Fig. S1. A collection of 45 vaginal lactobacilli was screened, and those strains showing better growth in MRS in 16 h (OD_600nm_ >1) were selected for further characterization (data not shown). The ability of 28 strains (15 *L*. *gasseri* and 13 *L*. *crispatus*) to inhibit the growth of four common yeast and bacterial pathogens involved in VVC and BV was investigated. Supernatants of *L. gasseri* and *L. crispatus* strains tested in broth inhibition assays showed different degrees of inhibition against *Candida* spp. strains (Table S6). Six strains displayed >60% inhibition, and 10 strains displayed between 30% and 60% inhibition against one of the two *Candida* spp. strains. Agar spot test results showed that probiotic candidates were overall more active against *P. bivia*, with nine strains generating an inhibitory halo of 11–19 mm radius, than against *G. vaginalis*, with all the strains exerting a moderate inhibition (1–10 mm inhibitory radius).

As a result of growth curve and antagonistic experiments, seven strains (six *L*. *gasseri* and one *L*. *crispatus*) were selected, and their resistance to simulated gastrointestinal conditions was subsequently evaluated *in vitro* (Table S7). Those strains showing a log10 CFU reduction greater than 0.1 in the pH 2.3 experiment and 2.5 in the bile acid stress test were discarded from further analyses.

The adhesion capacity to vaginal HeLa cells of the remaining four candidates (three *L*. *gasseri* and one *L*. *crispatus*) was investigated as the final criterion for the selection of probiotic candidates. *L. crispatus* CECT 30647 showed the best adhesion capacity to vaginal epithelial cells followed by *L. gasseri* CECT 30648 (Table 1), and they were therefore selected for further *in vitro* characterization and clinical trial.

**TABLE 1:** Adhesion capacity of *L. crispatus* and *L. gasseri* final candidates to HeLa cells expressed in percentage ± SD^*a*^

Strain	Adhesion (%)	SD
*L. crispatus* CECT 30647	80.5	2.2
*L. gasseri* CECT 30648	74.7	3.3
*L. gasseri* 12-07	72.5	0.2
*L. gasseri* 12-05	74.6	0.1

^*a*^ SD, standard deviation.

Our *in silico* approach led to the detection of five potential adhesins (containing both cell wall anchor domains and YSIRK signals) in *L. crispatus* CECT 30647 genome and nine in *L. gasseri* CECT 30648 genome (Table S8). Only two potential adhesins presented mucus-binding domains (three in total) in the *L. crispatus* genome, whereas in *L. gasseri*, seven out of nine potential adhesins showed a variable but larger number of mucin domains, with proteins such as MEW1746773.1 displaying up to 17 Muc_BP or Muc_B2 domains or MEW1747461.1 containing 12 mucus-binding domains. Of note, extremely large adhesins were predicted for *L. gasseri*, such as proteins MEW1746869.1 (4,370 aa), MEW1746773.1 (3,685 aa), or MEW1746279.1 (2,814 aa).

### Functional characterization of *L. gasseri* CECT 30648 and *L. crispatus* CECT 30647

To gain more insights into the probiotic potential in the context of vaginal health of the two selected candidates, additional dedicated experiments were performed.

#### Inhibition of pathogens associated with aerobic vaginitis, urinary tract infections, and negative obstetric outcomes

**Fig 2:**
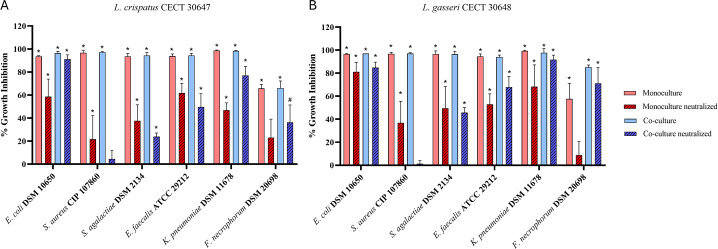
Antagonistic activity presented as percent growth inhibition of supernatants of (A) *L. crispatus* CECT 30647 and (B) *L. gasseri* CECT 30648 grown alone (monoculture) or in combination with the pathogen (co-culture) with or without pH neutralization. Statistical analyses were performed by the one-way analysis of variance with Dunnett’s multiple comparison test. *P < 0.0001, #*P* < 0.01.

Culture supernatants of *L. gasseri* CECT 30648 and *L. crispatus* CECT 30647 grown alone (monoculture) or in the presence of the pathogen (co-culture) with and without pH neutralization were tested for their antagonistic activity against pathogens causing AV and UTIs and involved in negative reproductive outcomes (Fig. 2).

*L. gasseri* CECT 30648 and *L. crispatus* CECT 30647 monoculture and co-culture supernatants showed almost complete inhibition (*P* < 0.0001) of all pathogens tested. pH neutralization retained the activity of both probiotic supernatants by 10%–90% except for co-culture samples against *S. aureus*, in which only marginal inhibition was observed. These results suggest that beyond production of fatty acids, both probiotic strains may also produce antimicrobial molecules that complement their inhibitory capacity. Interestingly, the inhibitory activity of *L. gasseri* strain against *Fusobacterium necrophorum* was markedly higher in neutralized co-culture supernatants than in neutralized monoculture samples. *In silico* analyses predicted seven potential bacteriocins in the genome of *L. crispatus* CECT 30647, including enterolysin A, penocin A, and helveticin J, and two bacteriocins were detected in the genome of *L. gasseri* CECT 30648 belonging to helveticin J and enterolysin A classes (Table S9). Also, production of lactate was confirmed in individual culture supernatants of *L. gasseri* CECT 30648 (12.8 g/L) and *L. crispatus* CECT 30647 (11.9 g/L) showing 45.4% and 35.6% L-lactic acid productions, respectively.

#### Resistance to vaginal tract

*L. gasseri* CECT 30648 tolerated better simulated vaginal medium conditions than *L. crispatus* CECT 30647 (*P* < 0.001), particularly after 24 h incubation (Table 2).

**TABLE 2:** Resistance of *L. gasseri* and *L. crispatus* strains to simulated vaginal medium expressed as log10 CFU/mL ± standard deviation

Strain	*T* = 0	*T* = 3	*T* = 6	*T* = 24	Log loss *T*0–*T*3	Log loss *T*0–*T*6	Log loss *T*0–*T*24
*L. gasseri* CECT 30648	5.48 ± 0.08	5.54 ± 0.08	5.49 ± 0.04	4.91 ± 0.16	−0.06 ± 0.11	−0.02 ± 0.1	0.57 ± 0.22
*L. crispatus* CECT 30647	5.39 ± 0.01	5.03 ± 0.05	3.94 ± 0.18	2.08 ± 0.11	0.36 ± 0.06	1.45 ± 0.19	3.32 ± 0.13

In addition, the ability of *L. gasseri* CECT 30648 and *L. crispatus* CECT 30647 to grow in the presence of the biogenic amines cadaverine and tyramine was investigated. Results showed that both strains can survive at all concentrations tested without significant differences compared to the growth in standard conditions (*P* > 0.05) (Fig. S3), indicating they can tolerate levels found in some conditions of vaginal dysbiosis. Also, we confirmed candidate strains cannot produce histamine, tyramine, putrescine, or cadaverine.

### Clinical trial

#### Participants

A total of 48 participants were enrolled in the study. At the end of the intervention, two participants were excluded from analysis due to lack of study compliance (low intake of study product), resulting in 46 participants included in the PP analyses. All 48 participants were included in the safety analysis since all had received at least one dose of the study product. Demographic and clinical characteristics, as well as time that elapsed since the last period, were similar among the groups (Table 3). There were no reports of lower abdominal pain, vaginal inflammation, dryness, itching, burning sensations, infertility, or vulvovaginal infections among the randomized participants. A total of 20 adverse events were reported (Table S10), of which 9 were related to digestive complaints (3 in each group), and 11 were related to other conditions (6 in Lg + Lc, 3 in Lg, and 2 in placebo). None of these events was rated as severe. No clinically relevant abnormalities were observed in glucose levels, renal function markers, liver function tests, lipid profile, electrolytes, hematological indices, or urinalysis parameters.

**TABLE 3:** Demographic characteristics of clinical study participants^*a*^

Characteristic	Lg + Lc (*n* = 18)	Lg (*n* = 16)	Placebo (*n* = 12)
Age (years)	25 .78 (5.55)	27.56 (6.55)	27.17 (6.91)
BMI (kg/m^2^)	23.01 (5.39)	23.27 (3.27)	23.04 (4.04)
Days from last period	8.44 (1.97)	9.11 (3.55)	7.92 (1.52)
History of vaginal infection (%)	4 (22.22)	3 (18.75)	2 (16.67)

^*a*^ Values are expressed as mean (±SD) except for history of vaginal infections in which data are expressed as number of participants and percentage in parenthesis. Sample size per group is indicated.

#### Detection of probiotic strains in vaginal samples

The primary endpoint of the clinical trial was to assess the ability of the probiotic strains, administered orally, to reach the vaginal tract. A total of 620 vaginal swabs were extracted and analyzed by qPCR using strain-specific primers. Oligo specificity was validated against a collection of 20 vaginal *L. gasseri* and *L. crispatus* strains, and its sensitivity was confirmed by spiking vaginal samples (Table S2 to S5). Forty-six volunteers provided vaginal samples until day 12, 45 volunteers until day 15, and 35 participants until day 18, coinciding with the start of a new menstrual period.

**Fig 3:**
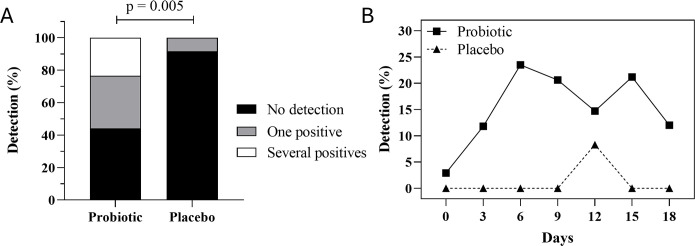
Detection of *L. gasseri* CECT 30648 during the study. (**A**) Overall percentage of volunteers that displayed qPCR positive results for *L. gasseri* CECT 30648 in probiotic (*L. gasseri* and *L. gasseri + L. crispatus* merged groups) and placebo groups. Black, percentage of volunteers with negative qPCR results; gray, percentage of volunteers with positive qPCR results obtained at 1 timepoint; white, percentage of volunteers with positive qPCR results obtained at >1 timepoint throughout the study. (**B**) Percentage of volunteers whose vaginal samples gave positive results at the different timepoints considered. Statistical analysis was performed comparing detection vs no detection with the chi-squared test.

According to qPCR experiments, *L. gasseri* CECT 30648 was detected in at least 1 out of the timepoints in 9 volunteers in the group receiving the strain alone (Lg) and in 10 volunteers in the group consuming the two-strain formula (Lg + Lc). Only one positive sample at day 12 was recorded in the placebo group throughout the study. *L. crispatus* CECT 30647 was not detected in any of the vaginal swabs at any timepoint. For this reason, the Lg and Lg + Lc groups were joined as a single group named probiotic for further analyses. Overall, *L. gasseri* CECT 30648 was detected in 19 out of 34 participants (55.9%) in the probiotic group and in 1 out of 12 (8.3%) in the placebo group (Table S11), the difference being statistically significant (*P* = 0.005) (Fig. 3A). Eleven volunteers displayed positive samples at one timepoint, four volunteers at two timepoints, and four volunteers at four timepoints. At day 3, four volunteers (11.8%) already had positive vaginal samples for *L. gasseri* (Fig. 3B). The number of participants with positive vaginal swabs increased the following days, peaking at day 6, with eight participants (23.5%) showing positive samples. The percentage of positive samples remained relatively stable (between ~15% and 22%) until day 18, when a slight decrease of positive swabs was noted.

#### Changes in vaginal microbiota composition and other secondary outcomes

**Fig 4:**
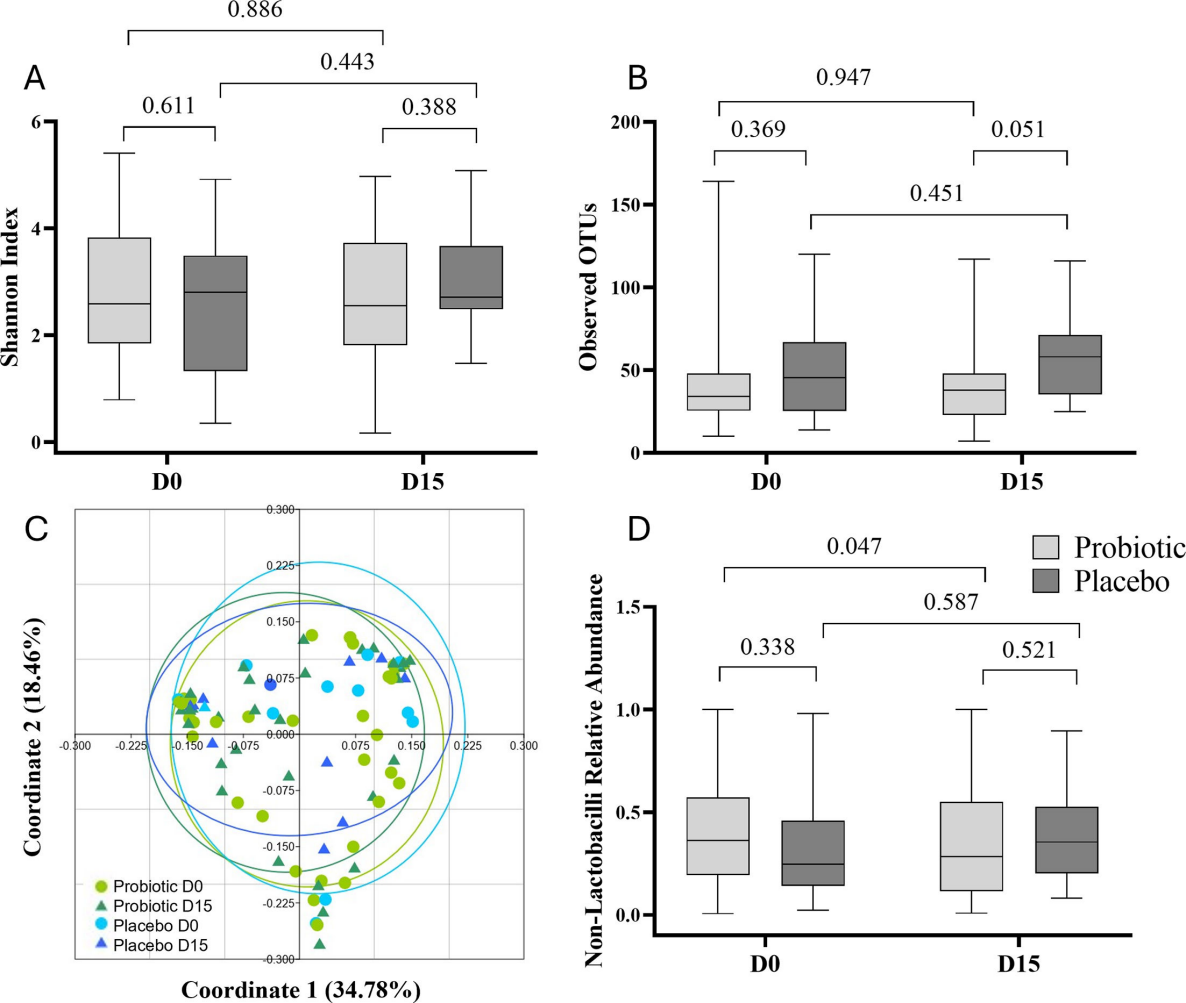
Microbiota analysis of vaginal samples studied by 16S rRNA sequencing. (**A**) Alpha-diversity measurements using Shannon index and (**B**) number of observed OTUs. (**C**) Beta-diversity by principal coordinate analysis (PCoA) plot with Bray-Curtis dissimilarity and (**D**) sum of relative abundances of non-lactobacilli genera of probiotic and placebo groups at baseline and day 15.

To assess vaginal microbiota composition, samples of all 45 participants from days 0 and 15 were considered, as at day 18, 10 participants already started a new menstrual cycle. A total of 90 samples from the probiotic and placebo groups were included in the analysis, and a total of 2,245 phylotypes were detected. No significant differences in alpha diversity or beta diversity were noted between groups or timepoints for any possible comparison as indicated by Shannon index and number of observed OTUs, and principal coordinate analysis plot with Bray-Curtis dissimilarity (Fig. 4A through C). No major changes were observed in individual non-lactobacilli genera. However, the sum of their relative abundances was reduced in the probiotic group at day 15 (*P* = 0.047 vs day 0), while this difference was not observed in the placebo group (*P* = 0.587) (Fig. 4D). In other words, *Lactobacillus* (*sensu lato*) relative abundance increased in the probiotic group over time. Of note, no differences in the relative abundances of the species *L. gasseri* or *L. crispatus* were observed in any comparison (*P* > 0.05, data not shown) by 16S rRNA sequencing.

Vaginal microbiota of participants were classified according to their CST (2) to investigate whether the different vaginotypes might influence probiotic colonization and vice versa. While the proportion of the CSTs in the placebo group remained stable during intervention, an increase of lactobacillus-dominated CSTs (mostly CST I) and a decrease in CST IV were noted in the probiotic group from days 0 to 15 (*P* = 0.039; Fig. S4).

Regarding other secondary outcomes, no significant differences in sexual hormone concentrations or vaginal pH were found between probiotic and placebo groups throughout the study period (Tables S12 and S13). Digestive tolerability to study products was also similar between the groups (data not shown).

## DISCUSSION

The use of oral probiotics for vaginal health is gaining interest because vaginal dysbiosis has been associated with multiple gynecological conditions (47). Probiotic therapy to improve vaginal health has traditionally used strains belonging to species of rare abundance in the vaginal ecosystem, either isolates from urogenital tract (15, 18), intestine (48), or fermented foods (49). Nowadays, healthy vaginal microbiotas are well defined (4), and the use of probiotics belonging to health-associated dominant species such as *L. crispatus* and *L. gasseri* is increasing (50–52).

The aim of this study was to characterize a collection of vaginal *L. gasseri* and *L. crispatus* isolates from the Kaneka collection to rationally select final candidates with the best potential attributes in the context of vaginal health and assess their vaginal colonization when administered orally in a randomized clinical trial in healthy women.

Our *in vitro* screening highlighted important phenotypic inter- and intraspecies variabilities among *L. gasseri* and *L. crispatus* vaginal isolates and allowed us to select the final candidates—*L. gasseri* CECT 30648 and *L. crispatus* CECT 30647—that, among many additional attributes, can resist simulated gastrointestinal conditions. This is a key feature for vaginal strains aimed to follow the oral-gut-vaginal route since it has been reported that vaginal *L. gasseri* and *L. crispatus* tend to show lower tolerance to gastrointestinal stress than intestinal isolates of the same species (53). As expected, selected strains, particularly *L. gasseri* CECT 30648, also showed resistance to simulated vaginal conditions. In addition, despite high levels of biogenic amines associated with vaginal dysbiosis that may inhibit the growth of certain lactobacilli (54), growth of *L. gasseri* CECT 30648 and *L. crispatus* CECT 30647 was not affected by the presence of these compounds, and none of the strains displayed biogenic amine production. This outcome supports the safety of the strains, together with the lack of transmissible antimicrobial genes described recently (30).

The two selected strains showed notable broad-spectrum antimicrobial activity against urogenital pathogens, including yeast and gram-positive and gram-negative bacteria, thus showing potential for the treatment of vaginal infections. Interestingly, we showed that *L. gasseri* CECT 30648 and *L. crispatus* CECT 30647 can inhibit the growth of *Fusobacterium* spp., a genus recently associated with negative pregnancy outcomes (13). Of note, the inhibition of the pathogen by *L. gasseri* was greater in neutralized co-culture samples than in neutralized monocultures, suggesting that the production of antimicrobials by the probiotic might be inducible. Antagonism against *Fusobacterium* spp. by *L .gasseri* has been reported for an oral isolate (55), but to our knowledge, this is the first time that a vaginal isolate shows inhibition against this relevant pathogen.

The final candidates, *L. gasseri* CECT 30648 and *L. crispatus* CECT 30647, were formulated in two different blends, and their colonization capacity after oral administration was studied in a population of healthy premenopausal women. The results of the placebo-controlled clinical study revealed that the two formulations (Lg and Lg + Lc) were well tolerated and demonstrated that *L. gasseri* CECT 30648 can colonize the vaginal tract in more than 55% of participants when supplemented daily between menstruations. On the contrary, *L. crispatus* CECT 30647 was not detected in any vaginal sample, which could be in part attributed to its lower resistance to bile salts observed *in vitro*. Also, *in silico* analyses revealed that *L. crispatus* CECT 30647 is poorly equipped with adhesion proteins containing LPxTG motifs, muc_B2/mucBP/mucBP_2 domains, and YSIRK signals, compared to Lg, which encodes for several adhesins, some of which are surprisingly large. It has been recently described that these adhesion determinants are positively selected in vaginal lactobacilli as they are essential for the interaction with the mucus of the vaginal epithelium (56).

*In vitro* adhesion experiments to HeLa cells (of cervical origin) probably represent a reductionist scenario of the real adhesion capacity of probiotics to different vaginal epithelial cells and mucins. This could contribute to explaining the apparent discrepancy between the similar *in vitro* adhesion ability of final candidates compared to their differential colonization *in vivo*. Finally, probiotic capsules contained lower *L. crispatus* dose than *L. gasseri* because the growth of the former during industrial production was lower than *L. gasseri*, compromising the feasibility of a potential product based on the *L. crispatus* CECT 30647 alone. Given the relevance of *L. crispatus* in vaginal health, the strain was nevertheless included at a lower dose according to the industrial production yield. It would be interesting to study if the production yield of *L. crispatus* in industrial media could be improved enough to consider a future clinical study with *L. crispatus* CECT 30647 as a single treatment at therapeutic doses.

Mixed results in terms of vaginal colonization have been reported for other *L. gasseri* and *L. crispatus* isolates, further highlighting that such ability is not only species specific but also strain specific. For instance, De Leo et al. (57) demonstrated that *L. crispatus* NTCVAG04 can reach the vagina after oral consumption, while Hertz et al. (58) and Qi et al. (50) showed *L. gasseri* DSM 14869, *L. gasseri* TM13, and *L. crispatus* LG55 cannot. Of note, *L. gasseri* TM13 and *L. crispatus* LG55 are of fecal origin, which may hamper their vaginal adaptation and colonization. Although *L. gasseri* DSM 14869 is a vaginal isolate, this probiotic was initially selected for vaginal administration, and thus, the gastrointestinal resistance was not assessed (59), and the origin of NTCVAG04 seems unpublished. Similar results have also been reported for strains belonging to other lactobacilli species (38, 60, 61), further corroborating that the oral-gut-vaginal route is a difficult journey only within reach of few adapted strains.

The study design allowed us to monitor the progression of the probiotic colonization. Interestingly, more than 10% of participants were already colonized by *L. gasseri* CECT 30648 at day 3, demonstrating a very rapid effect, whereas the peak of colonization (~15% to 24% of the study population) was observed between days 6 and 15. These results strongly suggest that *L. gasseri* CECT 30648 can survive gastrointestinal transit in humans and efficiently and rapidly colonize the vaginal tract. Importantly, to our knowledge, our study is the first to show a *L. gasseri* strain able to colonize the vaginal tract following oral administration likely by the oral-gut-vaginal route. Nevertheless, additional mechanisms such as immune cell trafficking, as the proposed enteromammary pathway hypothesis (62), cannot be discarded and deserve further research.

Our probiotic intervention exerted minor but interesting changes in the vaginal microbiota of healthy participants. In accordance, Koirala and co-workers (60) also reported slight microbiota changes by 16S metagenomics in a similar study in a healthy population. Nevertheless, our analysis aggregating the relative abundances of non-lactobacilli genera revealed that only participants in the probiotic group showed a significant reduction of the sum at day 15 compared to baseline, while this did not occur in the placebo group. In this line, further CST analyses also revealed interesting insights. Although our study population was healthy as per inclusion criteria, at the beginning of the intervention, 13 participants displayed CST IV, which is characterized by a lower level of lactobacilli and a higher level of anaerobic bacteria and has been associated with vaginal dysbiosis and BV. Interestingly, the most prevalent vaginotype among volunteers was CST III (17 participants), which is dominated by *L. iners* and, in some instances, has been suggested to predispose to CST IV (63, 64) and preterm delivery (65, 66). Thirteen volunteers showed a healthy *L. crispatus*-dominated CST I; only 1 participant qualified as CST II (*L. gasseri*-dominated microbiota), and 1 participant displayed *L. jensenii*-dominated CST V. Vaginal microbiotas dominated by *L. iners* have been recently observed in an Italian cohort (67), in contrast to Belgian, Swedish, and Danish populations (5, 68), suggesting this might be a typical trait of southern European women.

Interestingly, the number of CST IV participants in the probiotic group was reduced after intervention in favor of CST I, while CST II and III proportions remained stable. Of note, despite not reaching statistical significance, *L. gasseri* CECT 30648 was detected in 8 out of 11 (73%) participants showing CST IV and in 10 out of 22 (45%) volunteers with CST I, II, III, or V at day 0. Nonetheless, *L. gasseri* CECT 30648 detection did not translate into an increased prevalence of CST II on day 15, perhaps because probiotic presence and intervention were too short to become abundant in the vaginal environment. Also, it cannot be discarded that genome extraction process and 16S amplification might introduce a bias underestimating *L. gasseri* presence (69). Of note, a transition toward CST I was noted in three participants in the Lg group and in two participants in the Lg + Lc group, thus discarding the possibility to correspond to *L. crispatus* CECT 30647 presence.

Taken together, these results suggest that *L. gasseri* CECT 30648 colonization is enhanced in a dysbiotic environment and that the probiotic could compete and displace potentially detrimental bacteria in the vaginal tract. Further studies should consider increasing the number of participants, extending the intervention period, and testing different probiotic doses to determine if the favorable microbiota shift is due to direct or indirect probiotic action or rather due to other hormonal and menstrual cycle-related effects, which have been shown to influence vaginal microbiota composition (67). Likewise, additional efforts should be dedicated to validating the positive effects of *L. gasseri* CECT 30648 therapy in conditions of vaginal infection and its application in reducing gynecological disorders.

The main strengths of the clinical study include a close monitoring of colonization with sampling every 3 days, a randomized double-blind placebo-controlled design, and the use of a reliable detection method. Vaginal colonization was evaluated in a total of seven sampling points over a supplementation period of 18 days. The significance of conducting placebo-controlled trials is highlighted in our study since one subject at day 0 in the probiotic group and one subject at day 12 in the placebo group gave positive qPCR amplification. Indeed, literature has evidenced the importance of including a control group in this type of study. In 2001, Reid et al. (18) reported that *Lacticaseibacillus rhamnosus* GR-1 and *Limosilactobacillus fermentum* RC-14 were found in the vagina of all 10 patients treated orally in a non-controlled study. However, further trials with larger sample sizes and placebo-controlled designs showed low colonization rates of the same strains without differences with the control group (61, 70).

Notably, different detection techniques may yield contradictory results, and the use of a reliable method is essential. For example, *Lactobacillus acidophilus* La-14 and *L. rhamnosus* HN001 have been reported to reach the vagina since an increase of the species levels was observed by species-specific qPCR analysis (37, 38). However, these results could not be replicated when using strain-specific qPCR (71), and it was noted that primers used previously to detect *L. acidophilus* amplified other lactobacilli species.

To detect the strains in our study, we developed a strain-specific qPCR method. While qPCR allowed detection of the presence of *L. gasseri* strain, 16S metagenomics data showed no changes in the relative abundance of the species, indicating strain-specific qPCR is a more sensible technique for this purpose. This points out that negative colonization results that only rely on 16S rRNA sequencing (61) might not be accurate. Although we efficiently validated the specificity of the primers also by spiking in different independent vaginal samples, we observed the presence of a highly similar and resident *L. gasseri* strain in two samples, and this is a limitation. As it is impossible to ensure that a natural bacterial DNA region is only present in one specific strain in nature, future studies combining strain-specific qPCR with deeper sequencing strategies, such as shotgun analysis, should be conducted to gain resolution on the real probiotic colonization and microbiota modulation. Nonetheless, the combination of a sensitive detection method with a placebo-controlled study design and an unequivocally statistical significance (*P* = 0.005 probiotic vs placebo) leads us to conclude that oral probiotic intervention resulted in a true *L. gasseri* CECT 30648 vaginal colonization.

In conclusion, we show here that a well-designed *in vitro* screening, including key experiments of resistance to gut and vaginal environments using vaginal isolates of dominant species, is fundamental to selecting a successful strain able to colonize the vaginal tract through the oral-gut-vaginal route. Our strategy allowed us to identify the probiotic strain *L. gasseri* CECT 30648, which can colonize the vaginal tract of healthy women after oral consumption. Together with a broad-spectrum antimicrobial activity, these results position *L. gasseri* CECT 30648 as a promising probiotic that can be effective in promoting vaginal health.

## Data Availability

The 16S rRNA sequencing data generated in this study are available in the Sequence Read Archive. The associated BioProject number is PRJNA1286801.

## SUPPLEMENTAL MATERIAL

Supplemental tables and figures spectrum.00211-25-s0001.docx Tables S1 to S13 and Figures S1 to S4.

ASM does not own the copyrights to Supplemental Material that may be linked to, or accessed through, an article. The authors have granted ASM a non-exclusive, world-wide license to publish the Supplemental Material files. Please contact the corresponding author directly for reuse.
