# Supplementary material for: Lactobacillus gasseri CECT 30648 shows probiotic characteristics and colonizes the vagina of healthy women after oral administration
Source: Microbiol Spectr. 2025 Aug 7;13(9):e00211-25. doi: 10.1128/spectrum.00211-25 (PMC12403894; doi:10.1128/spectrum.00211-25)
Supplement: Supplemental tables and figures — Tables S1 to S13 and Figures S1 to S4. [file spectrum.00211-25-s0001.docx]

***Lactobacillus gasseri* CECT 30648 shows probiotic characteristics and colonizes the vagina of healthy women after oral administration**

Marta Perez^1#^, Eva Armengol^1,2#^, Antonio Del Casale ^3^, Ilenia Campedelli ^3^, Ana Aldea-Perona ^4,5,6^, Marta Pérez Otero ^4,5^, Maria Rodriguez-Palmero^1^, Jordi Espadaler-Mazo^1^ and Pol Huedo^1,2*^

1 R&D Department, AB-Biotics S.A. (Part of Kaneka Corporation), Barcelona, Spain,

2 Basic Sciences Department, Universitat Internacional de Catalunya, Barcelona, Spain

3 MICROBION srl, Open Innovation Department, Verona, Italy

4 Clinical Pharmacology, Hospital del Mar, Barcelona, Spain

5 Hospital del Mar Research Institute, Barcelona, Spain

6 Universitat Pompeu Fabra, Barcelona, Spain

# These authors contributed equally.

*Corresponding author: Pol Huedo huedo@ab-biotics.com

**Supplemental Tables**

**Table S1.** Strains, origin and growth conditions used in this study.

| **Strain** | **Origin** | **Growth conditions** |
| --- | --- | --- |
| *Lactobacillus gasseri* 12-01 | Healthy woman vagina | MRS, anaerobiosis, 37ºC |
| *L. gasseri* 12-04 | Healthy woman vagina | MRS, anaerobiosis, 37ºC |
| *L. gasseri* 12-05 | Healthy woman vagina | MRS, anaerobiosis, 37ºC |
| *L. gasseri* 12-07 | Healthy woman vagina | MRS, anaerobiosis, 37ºC |
| *L. gasseri* 13-01 | Healthy woman vagina | MRS, anaerobiosis, 37ºC |
| *L. gasseri* 18-02 | Healthy woman vagina | MRS, anaerobiosis, 37ºC |
| *L. gasseri* 20-02 | Healthy woman vagina | MRS, anaerobiosis, 37ºC |
| *L. gasseri* 22-16 | Healthy woman vagina | MRS, anaerobiosis, 37ºC |
| *L. gasseri* 22-17 | Healthy woman vagina | MRS, anaerobiosis, 37ºC |
| *L. gasseri* 28-11 | Healthy woman vagina | MRS, anaerobiosis, 37ºC |
| *L. gasseri* 31-02 | Healthy woman vagina | MRS, anaerobiosis, 37ºC |
| *L. gasseri* 31-13 | Healthy woman vagina | MRS, anaerobiosis, 37ºC |
| *L. gasseri* 31-18 | Healthy woman vagina | MRS, anaerobiosis, 37ºC |
| *L. gasseri* 31-22 | Healthy woman vagina | MRS, anaerobiosis, 37ºC |
| *L. gasseri* 35-28 | Healthy woman vagina | MRS, anaerobiosis, 37ºC |
| *L. gasseri* 40-13 | Healthy woman vagina | MRS, anaerobiosis, 37ºC |
| *L. gasseri* CECT 30648 (KABP064^TM^) | Healthy woman vagina | MRS, anaerobiosis, 37ºC |
| *L. gasseri* 43-13 | Healthy woman vagina | MRS, anaerobiosis, 37ºC |
| *L. gasseri* 44-01 | Healthy woman vagina | MRS, anaerobiosis, 37ºC |
| *L. gasseri* 47-08 | Healthy woman vagina | MRS, anaerobiosis, 37ºC |
| *Lactobacillus crispatus* 14-03 | Healthy woman vagina | MRS, anaerobiosis, 37ºC |
| *L. crispatus* 15-03 | Healthy woman vagina | MRS, anaerobiosis, 37ºC |
| *L. crispatus* 17-21 | Healthy woman vagina | MRS, anaerobiosis, 37ºC |
| *L. crispatus* 20-09 | Healthy woman vagina | MRS, anaerobiosis, 37ºC |
| *L. crispatus* 21-01 | Healthy woman vagina | MRS, anaerobiosis, 37ºC |
| *L. crispatus* 23-03 | Healthy woman vagina | MRS, anaerobiosis, 37ºC |
| *L. crispatus* 24-07 | Healthy woman vagina | MRS, anaerobiosis, 37ºC |
| *L. crispatus* 25-01 | Healthy woman vagina | MRS, anaerobiosis, 37ºC |
| *L. crispatus* 25-04 | Healthy woman vagina | MRS, anaerobiosis, 37ºC |
| *L. crispatus* 26-01 | Healthy woman vagina | MRS, anaerobiosis, 37ºC |
| *L. crispatus* 26-08 | Healthy woman vagina | MRS, anaerobiosis, 37ºC |
| *L. crispatus* 26-16 | Healthy woman vagina | MRS, anaerobiosis, 37ºC |
| *L. crispatus* 26-20 | Healthy woman vagina | MRS, anaerobiosis, 37ºC |
| *L. crispatus* 32-01 | Healthy woman vagina | MRS, anaerobiosis, 37ºC |
| *L. crispatus* 32-23 | Healthy woman vagina | MRS, anaerobiosis, 37ºC |
| *L. crispatus* 35-05 | Healthy woman vagina | MRS, anaerobiosis, 37ºC |
| *L. crispatus* 38-01 | Healthy woman vagina | MRS, anaerobiosis, 37ºC |
| *L. crispatus* CECT 30647 (KABP066^TM^) | Healthy woman vagina | MRS, anaerobiosis, 37ºC |
| *L. crispatus* 39-07 | Healthy woman vagina | MRS, anaerobiosis, 37ºC |
| *L. crispatus* 42-03 | Healthy woman vagina | MRS, anaerobiosis, 37ºC |
| *L. crispatus* 42-06 | Healthy woman vagina | MRS, anaerobiosis, 37ºC |
| *L. crispatus* 43-02 | Healthy woman vagina | MRS, anaerobiosis, 37ºC |
| *L. crispatus* 43-11 | Healthy woman vagina | MRS, anaerobiosis, 37ºC |
| *L. crispatus* 46-11 | Healthy woman vagina | MRS, anaerobiosis, 37ºC |
| *L. crispatus* 45-01 | Healthy woman vagina | MRS, anaerobiosis, 37ºC |
| *Candida glabrata* DSM 11226 | Blood | YPD, aerobiosis, 37ºC |
| *Candida albicans* DSM 1386 | Broncho mycosis | YPD, aerobiosis, 37ºC |
| *Prevotella bivia* DSM 20514 | Endometrium | Nutrient, aerobiosis, 37ºC |
| *Gardnerella vaginalis* DSM 4944 | Vaginal secretions | Nutrient, aerobiosis, 37ºC |
| *Escherichia coli UPEC* DSM 10650 | Clinical isolate from urine | TSB, aerobiosis, 37ºC |
| *Klebsiella pneumoniae* DSM 11678 | Human urinary tract | TSB, aerobiosis, 37ºC |
| *Fusobacterium necrophorum* DSM 20698 | Bovine liver abscess | BHI, anaerobiosis, 37ºC |
| *Staphylococcus aureus* CIP 107860 | Human skin | TSB, aerobiosis, 37ºC |
| *Streptococcus agalactiae DSM 2134* | Milk | BHI, anaerobiosis, 37ºC |
| *Enterococcus faecalis* ATCC 29212 | Urine | TSB, aerobiosis, 37ºC |

*MRS:* *De Man–Rogosa–Sharpe; YPD:* *Yeast Extract Peptone Dextrose; TSB: Trypticase Soy Broth; BHI: Brain Heart Infusion; CECT, Spanish Type Culture Collection; DSM, German Collection of Microorganisms; CIP, Institute Pasteur Collection; ATCC, American Type Culture Collection; UPEC, Uropathogenic Escherichia coli.*

**Table S2**. Genomes used in the identification of exclusive regions in *L. gasseri* CECT 30648 and *L. crispatus* CECT 30647 genomes.

| **Database** | **Species** | **Strain** | **Num of contigs** | **Accession number** |
| --- | --- | --- | --- | --- |
| *L. crispatus DB* | *L. crispatus* | CECT 30647 | 154 | JBFCOQ000000000 |
|  | *L. crispatus* | B4 | 1 | CP059140.1 |
|  | *L. crispatus* | CO3MRSI1 | 1 | NZ_CP033426.1 |
|  | *L. crispatus* | PRL2021 | 1 | NZ_CP058996.1 |
|  | *L. crispatus* | lc31 | 1 | NZ_CP061006.1 |
|  | *L. crispatus* | lc83 | 1 | NZ_CP061005.1 |
|  | *L. crispatus* | ATCC 33820 | 1 | NZ_CP072197.1 |
|  | *L. crispatus* | KT-11 | 1 | AP025162.1 |
|  | *L. crispatus* | Lc1226 | 1 | NZ_CP083392.1 |
|  | *L. crispatus* | Lc1700 | 1 | CP083389.1 |
|  | *L. crispatus* | PMC201 | 1 | NZ_CP076522.1 |
|  | *L. crispatus* | 43_08 | 212 | N/A |
| *L. gasseri DB* | *L. gasseri* | CECT 30648 | 7 | JBFCOP000000000 |
|  | *L. gasseri* | HL70 | 1 | NZ_CP072657.1 |
|  | *L. gasseri* | HL75 | 1 | NZ_CP071801.1 |
|  | *L. gasseri* | HL20 | 1 | NZ_CP072178.1 |
|  | *L. gasseri* | Lg1199 | 1 | NZ_CP087959.1 |
|  | *L. gasseri* | Lg1266 | 1 | NZ_CP087761.1 |
|  | *L. gasseri* | Lg637 | 1 | NZ_CP087763.1 |
|  | *L. gasseri* | 4M13 | 1 | NZ_CP021427.1 |
|  | *L. gasseri* | EJL | 1 | NZ_CP054875.1 |
|  | *L. gasseri* | 35_28 | 7 | N/A |
|  | *L. gasseri* | 12_07 | 7 | N/A |
|  | *L. gasseri* | 15_05 | 7 | N/A |

*N/A: Not available; DB: database*

**Table S3**. Strain-specific primers designed for *L. gasseri* CECT 30648 and *L. crispatus* CECT 30647 genomes.

| **Name** | **Sequence (5'-3')** | **N-mer** | **Tm** | **Amplicon length** | **Target strain** |
| --- | --- | --- | --- | --- | --- |
| LG4107_F1 | TGG GCA CTA GGT AAT AAG AAC | 21 | 62.1 | 119 | *L. gasseri*  CECT 30648 |
| LG4107_R1 | GGG TGT CCA GAT ATA TAT CCA | 21 | 62.3 |  |  |
| LC3901_F6 | AT CGC CGC TAA TAT CTT CAT C | 22 | 69.3 | 126 | *L. crispatus*  CECT 30647 |
| LC3901_R6 | TGA GCG ATG ATG CAC TTG A | 19 | 68.7 |  |  |

**Table S4**. Specificity of strain-specific primers designed for *L. gasseri* CECT 30648 and *L. crispatus* CECT 30647 DNA against a collection of *L. gasseri* and *L. crispatus* strains using pure DNA (100 ng/µl).

| **Sample** | **Average Ct** |
| --- | --- |
| **Oligos LG4107_(F/R) targeting *L. gasseri* CECT 30648** | |
| *L. gasseri* 12-05 | >35 |
| *L. gasseri* 12-07 | >35 |
| *L. gasseri* 13-01 | >35 |
| *L. gasseri* 22-16 | >35 |
| *L. gasseri* 31-22 | >35 |
| *L. gasseri* 35-28 | >35 |
| *L. gasseri* 40-13 | >35 |
| *L. gasseri* CECT 30648 | 11.07 |
| *L. gasseri* 44-01 | >35 |
| *L. gasseri* LN40 | >35 |
| **Oligos LC3901 (F/R) targeting *L. crispatus* CECT 30647** | |
| *L. crispatus* 21-01 | >40 |
| *L. crispatus* 24-07 | >40 |
| *L. crispatus* 15-03 | >40 |
| *L. crispatus* 17-21 | >40 |
| *L. crispatus* CECT 30647 | 14.26 |
| *L. crispatus* 42-03 | >40 |
| *L. crispatus* 43-08 | >40 |
| *L. crispatus* 42-06 | >40 |
| *L. crispatus* 45-14 | >40 |
| *L. crispatus* IP174178 | >40 |

**Table S5**. Sensitivity of strain-specific primers designed for *L. gasseri* CECT 30648 and *L. crispatus* CECT 30647 DNA against spikes in vaginal swabs (taken from placebo volunteers).

| **Volunteer** | **Spike** | **Concentration (ng/****µl)** | **Ct mean** | **SD** |
| --- | --- | --- | --- | --- |
| **Oligos LG4107 (F/R) targeting *L. gasseri* CECT 30648** | | | | |
| **1** | Spike 1 | 1 | 20.36 | 0.21 |
|  | Spike 2 | 0.1 | 24.56 | 0.21 |
|  | Spike 3 | 0.01 | 29.91 | 0.26 |
|  | Spike 4 | 0.001 | 33.37 | 0.22 |
|  | No Spike | 0 | >36 | na |
| **2** | Spike 1 | 1 | 21.65 | 0.11 |
|  | Spike 2 | 0.1 | 25.91 | 0.16 |
|  | Spike 3 | 0.01 | 31.38 | 0.37 |
|  | Spike 4 | 0.001 | 34.69 | 0.46 |
|  | No Spike | 0 | >36 | na |
| **Oligos LC3901 (F/R) targeting *L. crispatus* CECT 30647** | | | | |
| **3** | Spike 1 | 1 | 21.39 | 0.13 |
|  | Spike 2 | 0.1 | 24.8 | 0.16 |
|  | Spike 3 | 0.01 | 28.57 | 0.13 |
|  | Spike 4 | 0.001 | 33.24 | 0.23 |
|  | No Spike | 0 | >36 | na |
| **4** | Spike 1 | 1 | 21.55 | 0.09 |
|  | Spike 2 | 0.1 | 25.05 | 0.16 |
|  | Spike 3 | 0.01 | 28.74 | 0.17 |
|  | Spike 4 | 0.001 | 32.61 | 0.17 |
|  | No Spike | 0 | >36 | na |
| **5** | Spike 1 | 1 | 21.52 | 0.07 |
|  | Spike 2 | 0.1 | 24.78 | 0.12 |
|  | Spike 3 | 0.01 | 27.8 | 0.03 |
|  | Spike 4 | 0.001 | 33.75 | 1.98 |
|  | No Spike | 0 | >36 | na |

**Table S6.** Antimicrobial activity of *L. gasseri* and *L. crispatus* strains. Activity against vaginal bacterial pathogens was analysed by agar spot test, and activity against *Candida* spp. strains by broth inhibition assay.

| **Strain** | ***Gardnerella vaginalis***  **DSM 4944** | ***Prevotella bivia***  **DSM 20514** | ***Candida albicans***  **DSM 1386** | ***Candida glabrata***  **DSM 11226** |
| --- | --- | --- | --- | --- |
| ***L. gasseri* 12-01** | + | + | - | - |
| ***L. gasseri* 12-04** | + | + | - | - |
| ***L. gasseri* 12-05** | + | + | +++ | +++ |
| ***L. gasseri* 12-07** | + | + | +++ | +++ |
| ***L. gasseri* 13-01** | + | + | - | - |
| ***L. gasseri* 18-02** | + | + | ++ | +++ |
| ***L. gasseri* 20-02** | + | + | ++ | - |
| ***L. gasseri* 22-16** | + | + | ++ | - |
| ***L. gasseri* 22-17** | + | + | - | - |
| ***L. gasseri* 31-22** | + | ++ | ++ | - |
| ***L. gasseri* 35-28** | + | ++ | +++ | +++ |
| ***L. gasseri* 40-13** | + | ++ | ++ | - |
| ***L. gasseri* CECT 30648** | + | ++ | +++ | +++ |
| ***L. gasseri* 43-13** | + | + | - | - |
| ***L. gasseri* 44-01** | + | + | ++ | - |
| ***L. crispatus* 14-03** | + | ++ | ++ | - |
| ***L. crispatus* 15-03** | + | ++ | + | - |
| ***L. crispatus* 17-21** | + | + | - | - |
| ***L. crispatus* 21-01** | + | ++ | ++ | + |
| ***L. crispatus* 23-03** | + | + | - | - |
| ***L. crispatus* 24-07** | + | + | - | - |
| ***L. crispatus* 26-16** | + | + | - | - |
| ***L. crispatus* 38-01** | + | + | - | - |
| ***L. crispatus* CECT 30647** | + | + | ++ | +++ |
| ***L. crispatus* 42-03** | + | ++ | - | - |
| ***L. crispatus* 42-06** | + | ++ | ++ | - |
| ***L. crispatus* 43-11** | + | + | - | - |
| ***L. crispatus* 45-01** | + | + | - | - |

*For agar spot test: −, no inhibition; +, radius between 1 and 10 mm; ++, radius between 11 and 19 mm; +++, radius > 20 mm. For broth inhibition assay: −, no inhibition; +, inhibition between 1-30%; ++, inhibition between 30-60%; +++, inhibition >60% based on relative optical density.*

**Table S7**. Tolerance of selected *L. gasser*i and *L. crispatus* strains to gastric solutions (pH 2.3 and pH 3) and bile salts (0.25% w/v)

| **Strain**​ | **Gastric stress pH 2.3**​ | | | **Gastric stress pH 3**​ | | | **Bile salts stress**​ | | |
| --- | --- | --- | --- | --- | --- | --- | --- | --- | --- |
|  | **Count** | | **Loss**​ | **Count** | | **Loss**​ | **Count** | | **Loss**​ |
|  | **t=0 min**​ | **t=30 min**​ | **t=0 min - t=30 min**​ | **t=0 min**​ | **t=90 min**​ | **t=0 min - t=90 min**​ | **t=0 min**​ | **t=3 h**​ | **t=3 h - t=0 min**​ |
| ***L. gasseri* 12-05​** | 6.61 ± 0.54​ | 6.57 ± 0.55​ | 0.04 ± 0.01​ | 6.61 ± 0.54​ | 6.5 ± 0.49​ | 0.11 ± 0.04​ | 6.3 ± 0.04​ | 4.26 ± 0.12​ | -2.04 ± 0.08​ |
| ***L. gasseri* 12-07​** | 7.12 ± 0.06​ | 7.11 ± 0.05​ | 0.01 ± 0​.01 | 7.12 ± 0.06​ | 7.1 ± 0.07​ | 0.02 ± 0.01​ | 7.02 ± 0.03​ | 4.82 ± 0.09​ | -2.20 ± 0.06​ |
| ***L. gasseri* 18-02​** | 6.45 ± 0.38​ | 6.34 ± 0.33​ | 0.11 ± 0.05​ | 6.45 ± 0.38​ | 6.41 ± 0.33​ | 0.04 ± 0.05​ | 5.64 ± 0.09​ | 2.03 ± 0.02​ | -3.61 ± 0.12​ |
| ***L. gasseri* 20-02​** | 6.59 ± 0.4​ | 6.38 ± 0.21​ | 0.21 ± 0.16​ | 6.59 ± 0.4​ | 6.39 ± 0.19​ | 0.2 ± 0.17​ | 6.19 ± 0.03​ | 2.82 ± 0.01​ | -3.37 ± 0.03​ |
| ***L. gasseri* 35-28​** | 6.77 ± 0.12​ | 6.64 ± 0.04​ | 0.13 ± 0.07​ | 6.77 ± 0.12​ | 6.51 ± 0.06​ | 0.26 ± 0.05​ | 6.44 ± 0.07​ | 3.92 ± 0.03​ | -2.52 ± 0.04​ |
| ***L. gasseri* CECT 30648** | 6.48 ± 0.12​ | 6.39 ± 0.2​ | 0.10 ± 0.06​ | 6.48 ± 0.12​ | 6.41 ± 0.13​ | 0.08 ± 0.01​ | 6.35 ± 0.05​ | 5.20 ± 0.03​ | -1.25 ± 0.08​ |
| ***L. crispatus* CECT 30647** | 5.45 ± 0.31​ | 5.39 ± 0.33​ | 0.07 ± 0.01​ | 5.45 ± 0.31​ | 5.39 ± 0.26​ | 0.06 ± 0.04​ | 5.50 ± 0.07​ | 4.04 ± 0.02​ | -1.46 ± 0.05​ |

*Data are presented as mean and standard deviation of log CFU/mL.*

**Table S8.** Potential adhesins containing LPXTG motif, YSIRK signal and Mucus-binding domains (muc_B2/mucBP/mucBP_2) identified in the genomes of *L. crispatus* and *L. gasseri* strains

| Strain | Accession | aa | LPXTG* | YSIRK* | Mub* |
| --- | --- | --- | --- | --- | --- |
| *L. crispatus* CECT 30647 | MEW1575044.1 | 300 | 1 | 1 | 0 |
| *L. crispatus* CECT 30647 | MEW1575045.1 | 970 | 1 | 1 | 2 |
| *L. crispatus* CECT 30647 | MEW1575864.1 | 949 | 1 | 1 | 0 |
| *L. crispatus* CECT 30647 | MEW1576190.1 | 444 | 1 | 1 | 0 |
| *L. crispatus* CECT 30647 | MEW1576191.1 | 344 | 1 | 1 | 1 |
| *L. gasseri* CECT 30648 | MEW1746279.1 | 2814 | 1 | 1 | 10 |
| *L. gasseri* CECT 30648 | MEW1746773.1 | 3685 | 1 | 1 | 17 |
| *L. gasseri* CECT 30648 | MEW1746868.1 | 482 | 1 | 1 | 0 |
| *L. gasseri* CECT 30648 | MEW1746869.1 | 4370 | 1 | 1 | 5 |
| *L. gasseri* CECT 30648 | MEW1747129.1 | 2453 | 1 | 1 | 7 |
| *L. gasseri* CECT 30648 | MEW1747394.1 | 1688 | 1 | 1 | 7 |
| *L. gasseri* CECT 30648 | MEW1747408.1 | 1547 | 1 | 1 | 1 |
| *L. gasseri* CECT 30648 | MEW1747409.1 | 1108 | 1 | 1 | 0 |
| *L. gasseri* CECT 30648 | MEW1747461.1 | 2005 | 1 | 1 | 12 |

* Number of repeats detected either by eggNOG annotation or InterProt scan

**Table S9**. Bacteriocins predicted in the genomes of *L. gasseri* CECT 30648 and *L. crispatus* CECT 30647 strains through Bagel4 analysis.

| **Genome** | **Contig** | **start** | **end** | **Class** |
| --- | --- | --- | --- | --- |
| *L. crispatus* CECT 30647 | 9 | 37619 | 48641 | Enterolysin_A |
| *L. crispatus* CECT 30647 | 105 | 0 | 4344 | Penocin_A |
| *L. crispatus* CECT 30647 | 74 | 0 | 9411 | Helveticin-J |
| *L. crispatus* CECT 30647 | 58 | 1 | 11143 | Helveticin_J |
| *L. crispatus* CECT 30647 | 26 | 11489 | 31489 | LAPs |
| *L. crispatus* CECT 30647 | 7 | 2549 | 23017 | Enterolysin_A |
| *L. gasseri* CECT 30648 | 1 | 800373 | 821354 | Helveticin_J |
| *L. gasseri* CECT 30648 | 1 | 74195 | 94678 | Enterolysin_A |

**Table S10.** Summary of adverse events registered during the study.

|  | **Lg + Lc**  (n=18) | **Lc**  (n=18) | **Placebo**  (n=12) |
| --- | --- | --- | --- |
| **Digestive events; n (%)** | 3 (16.7) | 3 (16.7) | 3 (25) |
| Mild |  |  |  |
| Flatulence | 2 | 0 | 1 |
| Abdominal distension | 0 | 2 | 0 |
| Abdominal pain or discomfort | 1 | 0 | 2 |
| Constipation | 0 | 1 | 0 |
| **Other conditions; n (%)** | 6 (33.3) | 3 (16.7) | 2 (16.7) |
| Mild |  |  |  |
| Respiratory infection  (i.e.. pharyngitis, cough) | 1 | 1 | 0 |
| Atopic dermatitis, exacerbation | 0 | 1 | 0 |
| Low back pain | 1 | 0 | 0 |
| Moderate |  |  |  |
| Respiratory infection  (i.e.. pharyngitis, cough) | 2 | 0 | 0 |
| Headache | 1 | 1 | 0 |
| Severe |  |  |  |
| Headache | 0 | 0 | 1 |
| Toothache | 1 | 0 | 0 |
| Conjunctivitis | 0 | 0 | 1 |

**Table S11.** Mean Cts of participants that showed at least 1 positive qPCR amplification (ct≤36) throughout the study using specific primers targeting *L. gasseri* CECT 30648.

| **Volunteer** | **Group** | **Mean Ct** | | | | | | |
| --- | --- | --- | --- | --- | --- | --- | --- | --- |
|  |  | **D0** | **D3** | **D6** | **D9** | **D12** | **D15** | **D18** |
| O100 | Probiotic | na | na | 34.1 | na | na | 33.1 | ns |
| O103 | Probiotic | 32.6 | na | na | na | na | na | na |
| O105 | Probiotic | na | na | na | 35.5 | na | na | na |
| O112 | Probiotic | na | na | na | 31.3 | 33.3 | na | na |
| O113 | Probiotic | na | na | 28.2 | na | 25.7 | 31.1 | 31.8 |
| O115 | Probiotic | na | na | na | na | 34.0 | na | na |
| O116 | Probiotic | na | 30.5 | na | 33.9 | na | 28.4 | 27.3 |
| O117 | Probiotic | na | na | na | na | 35.3 | ns | ns |
| O119 | Probiotic | na | na | na | na | na | 33.0 | na |
| O120 | Probiotic | na | 33.5 | na | na | na | 33.3 | na |
| O123 | Probiotic | na | na | 32.7 | 33.2 | na | na | ns |
| O127 | Probiotic | na | na | 35.7 | na | na | na | ns |
| O130 | Probiotic | na | 34.9 | na | na | na | na | na |
| O131 | Probiotic | na | na | 35.6 | na | na | na | na |
| O134 | Probiotic | na | na | 32.3 | na | na | na | na |
| O143 | Probiotic | na | 35.3 | na | na | na | na | na |
| O144 | Probiotic | na | na | 34.9 | 33.0 | na | 33.2 | 35.9 |
| O145 | Probiotic | na | na | na | 35.9 | na | na | ns |
| O146 | Probiotic | na | na | 35.2 | 24.4 | 24.4 | 25.7 | ns |
| O147 | Placebo | na | na | na | na | 31.5 | na | ns |
| na: no amplification | |  |  |  |  |  |  |  |
| ns: no sample available | | |  |  |  |  |  |  |

**Table S12**. Sexual hormone concentrations in serum of study participants.

|  | **Probiotic (Lg+Lc and Lg)** | | | **Placebo** | | |
| --- | --- | --- | --- | --- | --- | --- |
|  | **D0** | **D9** | **D18** | **D0** | **D9** | **D18** |
| **E2 (pg/mL)** | 101.2 (79.9) | 179.5 (151.9) | 109.6 (77.0) | 76.3 (53.7) | 207.9 (123.2) | 173.2 (103.1) |
| **FSH (UI/L)** | 4.5 (2.7) | 5.5(4.9) | 3.5 (2.2) | 5.5 (1.6) | 5.9 (2.4) | 3.4 (1.4) |
| **LH (UI/L)** | 8.5 (8.1) | 18.3 (19.2) | 6.5 (4.7) | 9.5 (4.7) | 21.9 (16.4) | 6.1 (3.6) |
| **P4 (ng/mL)** | 4.5 (6.7) | 5.9 (6.4) | 5.7 (6.6) | 2.5 (5.1) | 3.7 (7.3) | 11.0 (11.5) |

*E2: Estradiol: FSH; Follicle stimulating hormone; LH: Luteinizing hormone; P4: Progesterone.*

*Data are presented as mean and standard deviation*

**Table S13.** Vaginal pH of study participants.

|  | **D0** | **D3** | **D6** | **D9** | **D12** | **D15** | **D18** |
| --- | --- | --- | --- | --- | --- | --- | --- |
| **Probiotic**  **(Lg+Lc and Lg)** | 4.9 (0.5) | 4.8 (0.5) | 4.7 (0.5) | 4.7 (0.4) | 4.6 (0.5) | 4.8 (0.4) | 4.8 (0.7) |
| **Placebo** | 4.8 (0.7) | 4.7 (0.5) | 4.8 (0.7) | 4.7 (0.3) | 4.8 (0.6) | 4.7 (0.8) | 4.6 (0.3) |

*Data are presented as mean and standard deviation*

**Supplemental Figures**


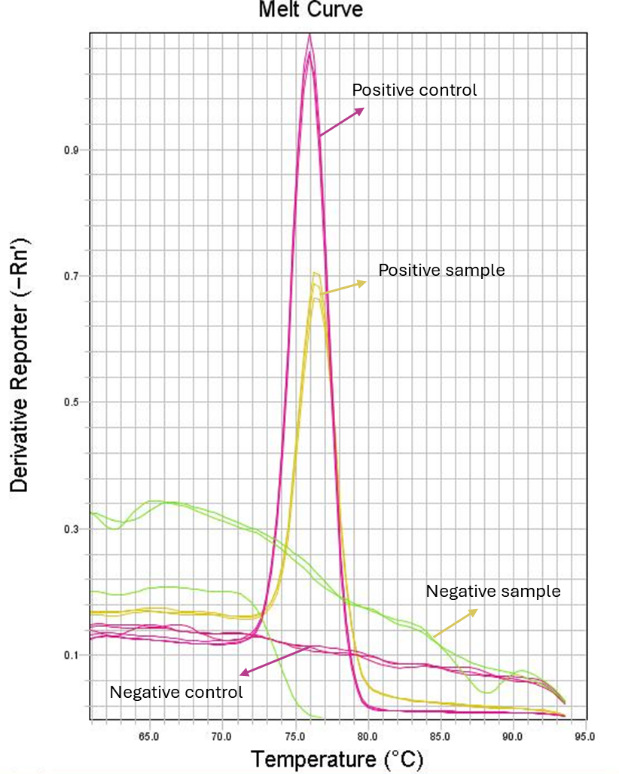


**Figure S1** Representative melt curves of qPCR positive and negative controls (pink), and positive (yellow) and negative (green) vaginal samples using primers LG4107_F1 and LG4107_R1.

**
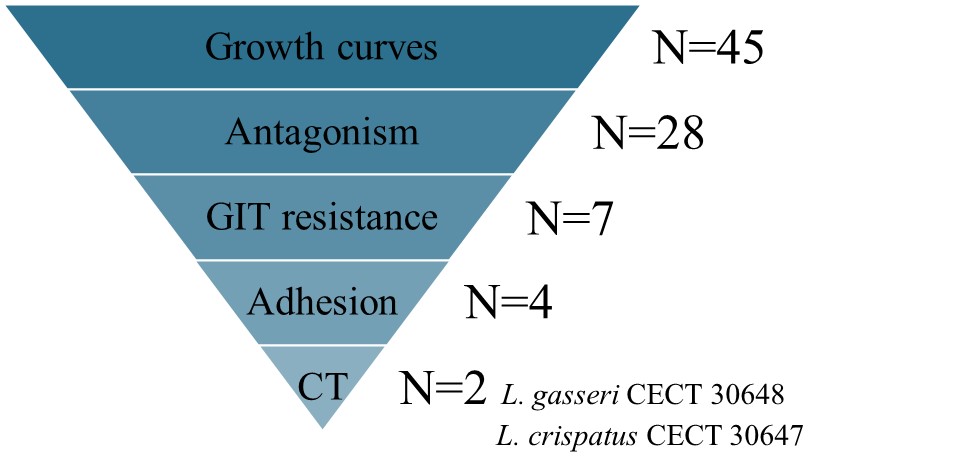
**

Figure S2. Schematic illustration of the strain selection process. GIT: gastrointestinal tract; CT: clinical trial.


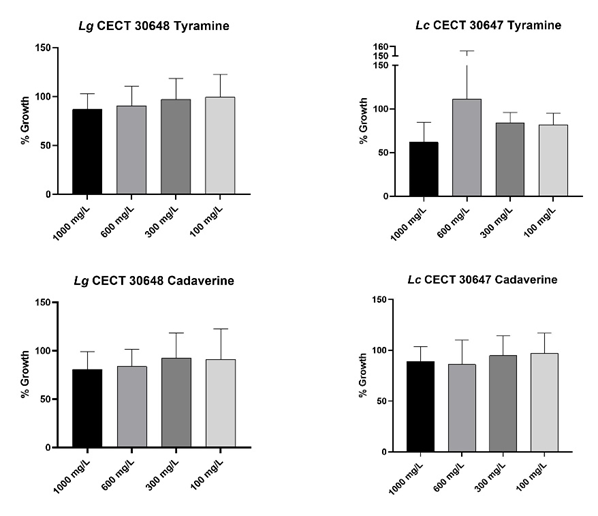


Figure S3. Growth percentage of *L. gasseri* and *L. crispatus* strains in the presence of different concentrations of tyramine and cadaverine relative to control condition without biogenic amines supplementation. Statistical analysis was performed with one-way ANOVA followed by Dunnet’s Multiple Comparison Test with no differences observed.


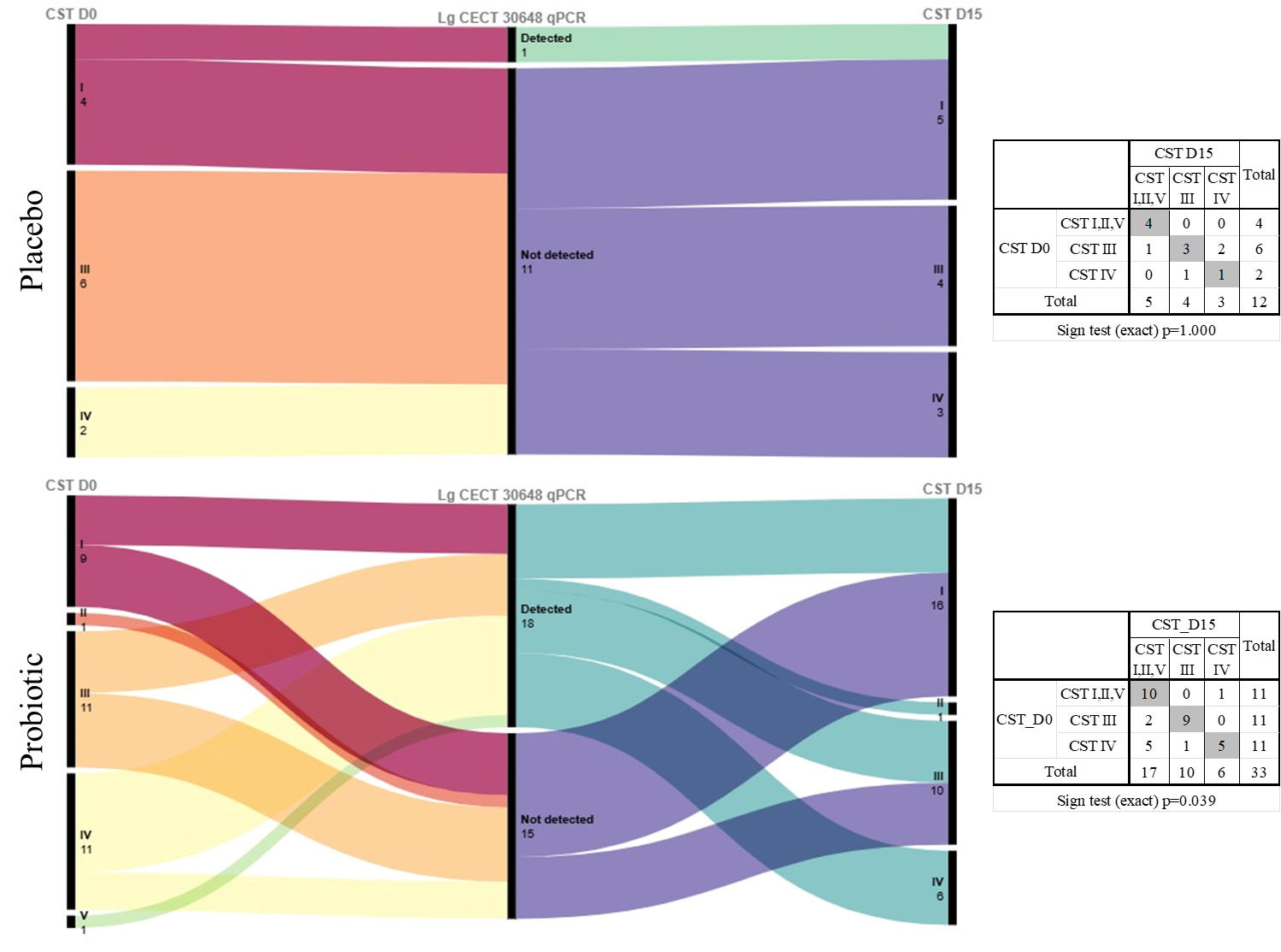


**Figure S4**. Alluvial plots of CST dynamics throughout the study in the full set of patients with available samples at day 0 and day 15 (n=45, 18 of which showed positive qPCR at least in one timepoint) in placebo (top) and probiotic (bottom) groups. The vaginal sample of one participant showing positive qPCR during days 0 to 12 was not available at day 15 and therefore was not included in the metagenomics and CST analysis. Statistical analysis was performed using sign (exact) test.
